# Clinical, Radiometabolic and Immunologic Effects of Olaparib in Locally Advanced Triple Negative Breast Cancer: The OLTRE Window of Opportunity Trial

**DOI:** 10.3389/fonc.2021.686776

**Published:** 2021-06-28

**Authors:** Francesco Schettini, Silvia Paola Corona, Fabiola Giudici, Carla Strina, Marianna Sirico, Ottavia Bernocchi, Manuela Milani, Nicoletta Ziglioli, Sergio Aguggini, Carlo Azzini, Giuseppina Barbieri, Valeria Cervoni, Maria Rosa Cappelletti, Alfredo Molteni, Maria Chiara Lazzari, Giuseppina Ferrero, Marco Ungari, Elena Marasco, Alice Bruson, Luciano Xumerle, Elisa Zago, Davide Cerra, Marco Loddo, Gareth H. Williams, Ida Paris, Giovanni Scambia, Daniele Generali

**Affiliations:** ^1^ Translational genomics and targeted therapies in solid tumors, August Pi I Sunyer Biomedical Research Institute (IDIBAPS), Barcelona, Spain; ^2^ Department of Clinical Medicine and Surgery, University of Naples Federico II, Naples, Italy; ^3^ Department of Medicine, Surgery and Health Sciences, University of Trieste, Cattinara Hospital, Trieste, Italy; ^4^ Multidisciplinary Unit of Breast Pathology and Translational Research, Cremona Hospital, Cremona, Italy; ^5^ Unit of Biostatistics, Epidemiology and Public Health, Department of Cardiac, Thoracic, Vascular Sciences and Public Health, University of Padua, Padua, Italy; ^6^ Unitá Operativa Ematologia e CTMO, Azienda Socio-Sanitaria Territoriale di Cremona, Cremona, Italy; ^7^ UO Anatomia Patologica ASST di Cremona, Cremona, Italy; ^8^ Personal Genomics Ltd, Verona, Italy; ^9^ Oncologica UK Ltd, Cambridge, United Kingdom; ^10^ Department of Life Sciences and Public Health, Università Cattolica del Sacro Cuore, Roma, Italy; ^11^ Department of Woman and Child Health, Fondazione Policlinico Universitario A. Gemelli IRCCS, Roma, Italy

**Keywords:** triple negative breast cancer, window of opportunity clinical trial, neoadjuvant, BRCA, olaparib (Lynparza™), TILs, PD-L1, homologous recombination deficiency

## Abstract

**Introduction:**

Olaparib is effective in metastatic triple negative breast cancer (TNBC) carrying germline mutations in DNA damage repair (DDR) genes *BRCA1/2* (g*BRCA*-mut). The OLTRE window-of-opportunity trial preliminarily investigated potential pathologic, radiometabolic and immune biomarkers of early-response to olaparib in g*BRCA*-wild-type (wt) TNBC and, as proof-of-concept in g*BRCA*-mut HER2-negative BC.

**Methods:**

Patients received olaparib for 3 weeks (3w) before standard neoadjuvant chemotherapy and underwent multiple FDG^18^-PET/CT scan (basal, after olaparib), clinical assessments (basal, every 3w), tumor biopsies and blood samplings (baseline, after olaparib). Clinical and radiometabolic responses were evaluated according to RECIST1.1 and PERCIST criteria.

**Results:**

27 patients with g*BRCA*-wt TNBC and 8 with g*BRCA*-mut BC (6 TNBC, 2 HR+/HER2-negative) were enrolled. Three (11.1%) patients showed mutations in non-*BRCA*1/2 DDR genes and 4 (14.8%) in other genes. 3w olaparib induced 16/35 and 15/27 partial clinical and radiometabolic responses, including in 40.7% and 50.0% g*BRCA*-wt patients. g*BRCA*-mut tumors presented numerically higher tumor-infiltrating lymphocytes (TILs) levels and PD-L1 positive tumors. Clinical responders experienced a reduction in T-regs/T-eff ratio (p=0.05), B and NK lymphocytes (p=0.003 both), with an average increase in T-helpers rate (p<0.001) and CD4/CD8 ratio (p=0.02). Ki67% and TILs did not vary significantly (p=0.67 and p=0.77). A numerical increase in PD-L1 positive cases after olaparib was observed, though non-significant (p=0.134). No differences were observed according to g*BRCA* status and type of response.

**Conclusions:**

Early-stage TNBC might be a target population for olaparib, irrespective of g*BRCA* mutations. Future trials should combine TILs, PD-L1 and g*BRCA* status to better identify candidates for escalated/de-escalated treatment strategies including olaparib.

## Introduction

Poly ADP-ribose polymerase (PARP) enzymes are critical for the repair of DNA single-strand breaks (SSB) and their disfunction favor SSB conversion into double-strand breaks (DSBs). If not repaired, the accumulation of DSBs can then lead to either cell death or neoplastic transformation (1, 2). The main mechanism of DSBs repair is represented by homologous recombination, though other mechanisms may intervene in dysfunctional cases (i.e. nonhomologous end joining and single-strand annealing) (1). An important cause for homologous recombination deficiency (HRD) is represented by hereditary germline mutations in the DNA repair genes *BRCA*1 or 2 (3). PARP-inhibitors (PARPi) are a novel drug class that proved to be effective in tumors harboring germline *BRCA1/2* (g*BRCA*) mutations, by inhibiting PARP enzymes and trapping PARP1 on the DNA, ultimately leading to cancer cell death (1, 4).

Triple Negative breast cancer (TNBC) is a heterogenous subgroup of prognostically unfavorable breast tumors, in urgent need for new personalized therapeutic approaches, as chemotherapy still remains their mainstay of treatment, due to the lack of well-defined molecular targets (5). In this perspective, a common characteristic of TNBC is the reduced expression of DNA damage repair (DDR) genes, with *BRCA1/2* being the most frequently affected (6). In fact, a mean 35% and 8% of TNBC are *BRCA1* and *BRCA2* mutant, respectively (7). The phase III trials OlympiAD (8) and EMBRACA (9) recently showed the superiority of the PARPi olaparib and talazoparib over standard chemotherapy in metastatic HER2-negative breast tumors (mostly TNBC) harboring germline *BRCA1/2 (gBRCA)* pathogenic variants, leading to their approval in this setting. While in ovarian cancer these agents demonstrated activity irrespective of the presence of a *BRCA* mutation (10, 11), it is still unclear if the use of PARPi could be extended to *BRCA*-wild type TNBC, as the few studies trying to address this question provided equivocal results (12–16). For this reason, we conducted the trial OLTRE. This study was a phase II, open label, single-center, window-of-opportunity (WoO) trial with olaparib administered as single agent for 3 weeks in locally advanced TNBC before standard neoadjuvant chemotherapy (17). Its purpose was to preliminarily detect potential pathological, radiometabolic and immune biomarkers of early response to olaparib, irrespective of g*BRCA* mutational status. As a proof of concept, analyses were performed also separately in a small group of g*BRCA*-mutant hormone receptor-positive (HR+) BC and TNBC, in which sensitivity to olaparib has already been proven (8).

Here we report the primary outcome and part of the secondary outcomes of the study.

## Materials and Methods

### Study Design and Eligibility Criteria

From September 2016 to July 2019, treatment-naïve patients with locally advanced BC (stage IIB-IIIC, according to the AJCC 7th edition, www.cancerstaging.org) undergoing neoadjuvant systemic therapy, with or without *BRCA* mutation, were enrolled at the ASST of Cremona Hospital in the OLTRE trial (Eudract 2015-000298-11) (17). Patients with HER2-positive (according to IHC and/or in-situ hybridization) BC, early-stage (TNM stage I-IIA) and metastatic tumors were excluded. Patients were divided into a subgroup of g*BRCA*-wild type TNBC and a subgroup of g*BRCA*-mutant HER2-negative BC. Patients were recruited if aged ≥18, with an Eastern Cooperative Oncology Group (ECOG) performance status <2 and with adequate baseline hematological, hepatic, renal and cardiac function. Full inclusion/exclusion criteria are reported in the study protocol (17).

The study was conducted in accordance with the Declaration of Helsinki, the Good Clinical Practice principles and all local regulations. The study obtained the approval of the ethical committee of the ASST of Cremona Hospital (IRB Approval 09/09/2015 n.21741/2015) and all participants provided written informed consent.

### Study Objectives and Endpoints

The main objective of the trial was to explore the biological effects of a short course of neoadjuvant olaparib in locally advanced HER2-negative BC, with a special focus on g*BRCA*-wild type TNBC. Secondary objectives included treatment activity, safety, tolerability and quality of life (QoL) and other correlative biomarker analyses.

The primary endpoint was the exploratory evaluation of the early changes induced by olaparib on several biomarkers, including Ki67, tumor-infiltrating lymphocytes (TILs), PD-L1 and circulating immune cells in the overall population enrolled.

Secondary endpoints included: (i) an exploratory assessment of the clinical and radiometabolic response rates in the overall population and according to *BRCA* mutational status; (ii) the correlation between *BRCA* status and changes in the pathologic, radiometabolic and immune markers; (iii) the study of the relationship occurring between baseline mutations, gene and protein expression profile and clinical response; (iv) exploratory tumor mutations analyses, including somatic *BRCA1/2* mutations, reversion mutations, loss of heterozygosity, genome landscape, transcriptional and functional measures of HRD; (v) the correlation of baseline mutations, gene and protein expression profile with PET/TC and/or clinical response after olaparib short course; (vi) the evaluation of safety and tolerability of olaparib alone assessed according to the Common Terminology Criteria for Adverse Events (CTCAE) version 4.03; (vii) the evaluation of time to deterioration of health-related quality of life by QLQ-C30 scale and the health status by QLQ-C30 scale.

QoL and gene/protein expression analyses will be documented separately.

### Study Treatment and Procedures

Patients were assigned to receive olaparib orally at a dose of 300mg (BD) on a continuous dosing regimen for 3 weeks (‘window therapy’) before undergoing standard neoadjuvant chemotherapy (anthracyclines and taxanes-based) and surgery. Tumor biopsies and blood sampling were performed at baseline and after 21 days of olaparib short course. Treatment stopped earlier if objective disease progression or unacceptable toxicity was detected. Toxicities were classified according to the (CTCAE) version 4.03.

FDG^18^-PET/CT scan was conducted at baseline and after 3 weeks of olaparib ± 3 days; contrast-enhanced breast magnetic resonance (MRI) and mammography (MMX) were conducted at baseline and before definitive surgery. Clinical assessments were conducted at baseline and every 3 weeks ± 3 days.

Clinical responses were evaluated through physical exam with caliper (18)and assessed according to RECIST1.1 criteria (19). The same operator performed all physical examinations pre/post olaparib short course therapy. Patients were categorized in two groups according to treatment response in responders (complete response + partial response [CR/PR]) and non-responders (stable disease + progressive disease [SD/PD]). Radiometabolic responsiveness to olaparib was measured by ^18^FDG-PET/CT. Patients were considered responsive to olaparib when a reduction of SUV_max_ was evident after 21 days, and not responsive when increase or stability in SUV_max_ was detected after 21 days of short course treatment. SUV response was defined according to PERCIST criteria (20). The same radiologist evaluated all PET/CT responses.

A pCR was defined as the absence of invasive breast tumor in the pathology specimen after surgery, both in breast and axilla, including the case of presence of residual *in situ* breast cancer (ypT0/is ypN0).

### Immunohistochemistry and TILs

Tissue from tumor specimens was obtained through biopsy of the breast lesion, fixed in paraffin and embedded in formalin (FFPE) for immunohistochemistry (IHC) analysis. Regions with non-invasive carcinoma, normal tissue or necrosis were excluded from the evaluation. Standard IHC was performed on FFPE for HER2, ER, PgR, and Ki67 staining using standard protocols and recommendations elsewhere described (21–23). Pathologists also scored 4–5 µm sections of FFPE tissues for the presence of stromal TILs. Tissues were scored as reported by Salgado et al (24) and, for descriptive purposes, classified into 3 categories according to their percentage, i.e. 0-10%, 10%<TILs<40% and TILs >40%-100%. Analyses were then conducted using TILs as continuous variable, as also suggested by Salgado et al (24).

### PD‐L1 SP142 CDx Immunohistochemistry Testing

Detection of Programmed Death-Ligand 1 (PD-L1) was performed on pre- and post-olaparib biopsy samples in tumor-infiltrating inflammatory cells. A cut-off of 1% was adopted to define negativity and positivity.

PD‐L1 IHC testing was conducted using the VENTANA SP142 CDx assay as *per* manufacturer’s instructions (**Supplementary Methods** for details). Tumor‐infiltrating inflammatory cells consisted of lymphocytes, macrophages, dendritic cells, and granulocytes. Tumor area for the purposes of this assay was defined as tumor cells and associated peritumoral and intratumoral stroma.

### Flow Cytometry Analysis

A study of circulating immune cells was performed on samples coming from the OLTRE Study. The whole blood samples before and after olaparib treatment allowed to analyze circulating lymphocytes and their changes under therapy. Flow cytometry analysis was performed with dual or triple-laser flow cytometers Becton Dickinson (BD) FACSCanto™ and BD FACSCanto II™, with BD™ Cytometer Setup and Tracking (CS&T) control, in order to make the signals reproducible and comparable regardless of the variation in environmental conditions. Acquisition of at least 1.5 x 10^6^ events was assessed by BDFACSC Diva software. The lymphocytes subpopulations (B, NK, T with CD4 and CD8 subpopulation) were assessed with BD Multitest 6-Color TBNK kit (Becton Dickinson™). More details are reported in **Supplementary Methods**.

### Mutational Status Analysis

All patients underwent germline genetic testing by a multi-gene panel including: (i) high-risk BC susceptibility genes such as *BRCA1, BRCA2, CDH1, PALB2, PTEN, STK11*, and *TP53;* (ii) moderate-risk BC susceptibility genes such as *ATM, BARD1, BRIP1, CHEK2, NBN, RAD51C*, and *RAD51D*; (iii) cancer predisposition genes related to other hereditary tumor syndromes such as *MLH1, MSH2, MSH6, PMS2, EPCAM, APC.* For this purpose, peripheral blood samples were collected from the enrolled patients; the analysis was assessed after the termination of treatment with olaparib, to be blinded to the response in the potential cohort of wild-type versus mutated patients. Genomic DNA was isolated from the peripheral blood using the DNeasy^®^ Blood Kit (QIAGEN), quantified by Qubit^®^ 3.0 fluorometer (Thermofisher Scientific, Waltham, MA, USA) and its quality was assessed by 2100 Bioanalyzer (Agilent Technologies, Santa Clara, CA, USA). 250 ng of DNA was used to prepare the barcoded library using Kapa Hyperplus kit (Roche). Target enrichment was performed using SeqCap EZ Choise kit (Roche) to perform a mutational screening on 19 genes involved in the risk of hereditary breast, ovarian and colorectal cancer, and other inherited tumor syndromes (*ATM, BARD1, BRCA1, BRCA2, BRIP1, CDH1, CHEK2, EPCAM, MLH1, MSH2, MSH6, NBN, PALB2, PMS2, PTEN, RAD51C, RAD51D, STK11, TP53, AXIN2, GALNT12* and *APC*). Sequencing was performed using the Illumina Miseq (Illumina). Genetic variants detected in the analyzed genes were validated by Sanger sequencing. Further details are reported in **Supplementary Methods**.

The interpretation of the clinical significance of the genetic variants identified was based on the classification criteria developed from the Evidence-based Network for the Interpretation of Germline Mutant Alleles (ENIGMA) consortium (https://enigmaconsortium.org/) and according to IARC recommendations (25). For the identification and classification of genetic variants, several databases, such as ClinVar, BRCA Exchange, LOVD, were used.

### Statistical Analyses

This study is exploratory and the sample size was not based on a formal statistical assumption, since no prior window study has provided data concerning olaparib short course in g*BRCA* wild-type TNBC and its impact on the potential biomarkers evaluated.

Continuous variables’ distribution was checked for normality with the Shapiro-Wilk test, then data were presented as mean and standard deviations (SD) if normally distributed, or median and range (minimum-maximum) if not. Parameters of interest (dimension, Ki67, SUV_max_, stromal TILs, circulating T-reg, relative percentage of circulating CD3+CD4+, CD3+CD8+, CD16+CD56+ and CD19+, CD3+CD4+ count, CD3+CD8+ count, CD4/CD8 ratio, CD19+ count, CD16+CD56+ count and PD-L1 status) before and after olaparib treatment were compared using Wilcoxon matched pairs test, t-student test for paired continuous data, McNemar test or Stuart-Maxwell test for paired categorical data, where appropriate.

Relative changes (Δ) in continuous variables were calculated using the formula: (post-olaparib values - pre-olaparib values)/pre-olaparib values. Parameters changes were investigated in terms of radiological and clinical response with the use of Mann-Whitney or t-student test for independent continuous data. Associations between PD-L1, mutational status and clinical/radiological response were assessed through χ^2^ test or Fischer Exact test (when appropriate). Statistical software R (version 4.0.0, 2020) was used for all analyses and a p value<0.05 was deemed to be significant, although formal comparisons were only exploratory.

## Results

### Patients and Tumors Characteristics

**Figure 1:**
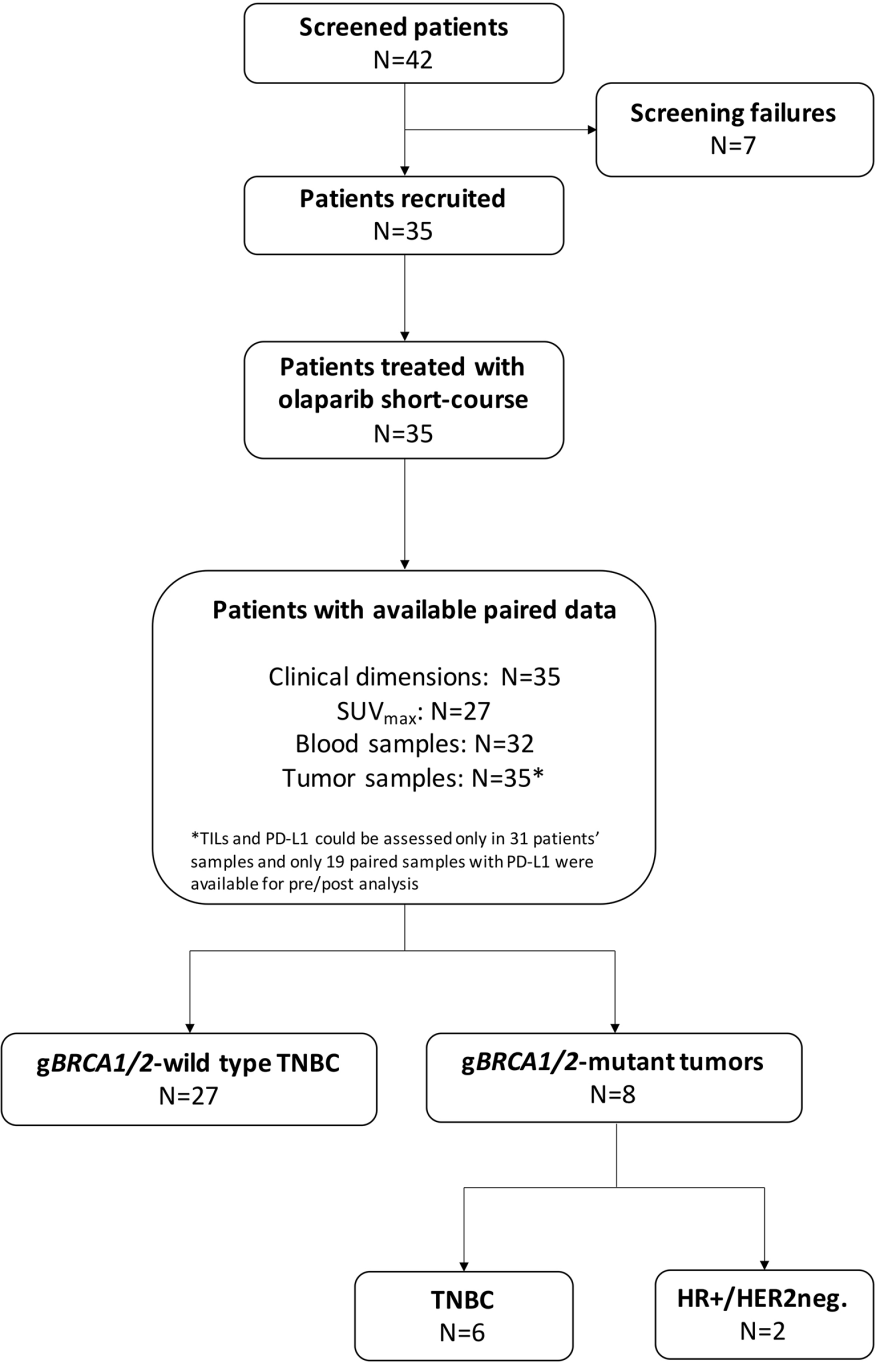
CONSORT diagram. TNBC, triple negative breast cancer; HR+, hormone receptor-positive; HER2neg., HER2-negative; g*BRCA1/2*, germline *BRCA1* and *BRCA2;* SUV, standard uptake volume; TILs, tumor-infiltrating lymphocytes.

A total of 35 patients were enrolled in the OLTRE trial (**Figure 1**). Twenty-seven patients (77.1%) carried TNBC without g*BRCA* mutations, while 2 (5.7%) patients carried g*BRCA*-mutant hormone receptor-positive (HR+)/HER2-negative BC and 6 (17.2%) g*BRCA*-mutant TNBC. Among *BRCA*-mutant patients, 7 (87.5%) presented a g*BRCA1* mutation deleterious or suspected to be deleterious, while 2 (12.5%) showed a germline mutation in *BRCA2*. Three (11.1%) patients showed mutations of genes involved in the DDR pathways (i.e. *BARD1, MSH2-3, RAD51C, PMS2* and *ATM*) and 4 (14.8%) carried mutations in genes other than the ones implicated in DDR (i.e. *PTEN, AXIN2, APC, GALNT12*), 2 of which of unknown clinical significance (**Supplementary Table 1**). Other 3 (11.1%) patients were not tested due to collection of inadequate quantity of germline DNA, while all other 17 (63.0%) patients did not show germline mutations detectable with the multigene panel.

Median clinical primary tumor dimension was 39mm (min-max: 18 – 80mm), mean PET/CT tumor dimension was 29.8mm (SD: ± 17.4mm), with a median SUV_max_ of 9.3 (min-max: 1.9 – 31.0) and a median Ki67 of 50% (min-max: 10 – 90%). Median circulating T-regs were 80 (12 – 297), while CD3+CD8+ (T suppressor) were 361 (144 – 932) and mean CD3+CD4+ (T helper) were 961 (SD: ± 451) with a mean CD4/CD8 ratio of 2.90 (SD: ± 1.61) and mean T-regs/T-effectors (T-eff) of 0.07 (SD: ± 0.03). Mean CD19+ (B lymphocytes) and CD16+CD56+ (NK lymphocytes) were 226 (SD: ± 139) and 367 (SD: ± 182), respectively.

**Figure 2:**
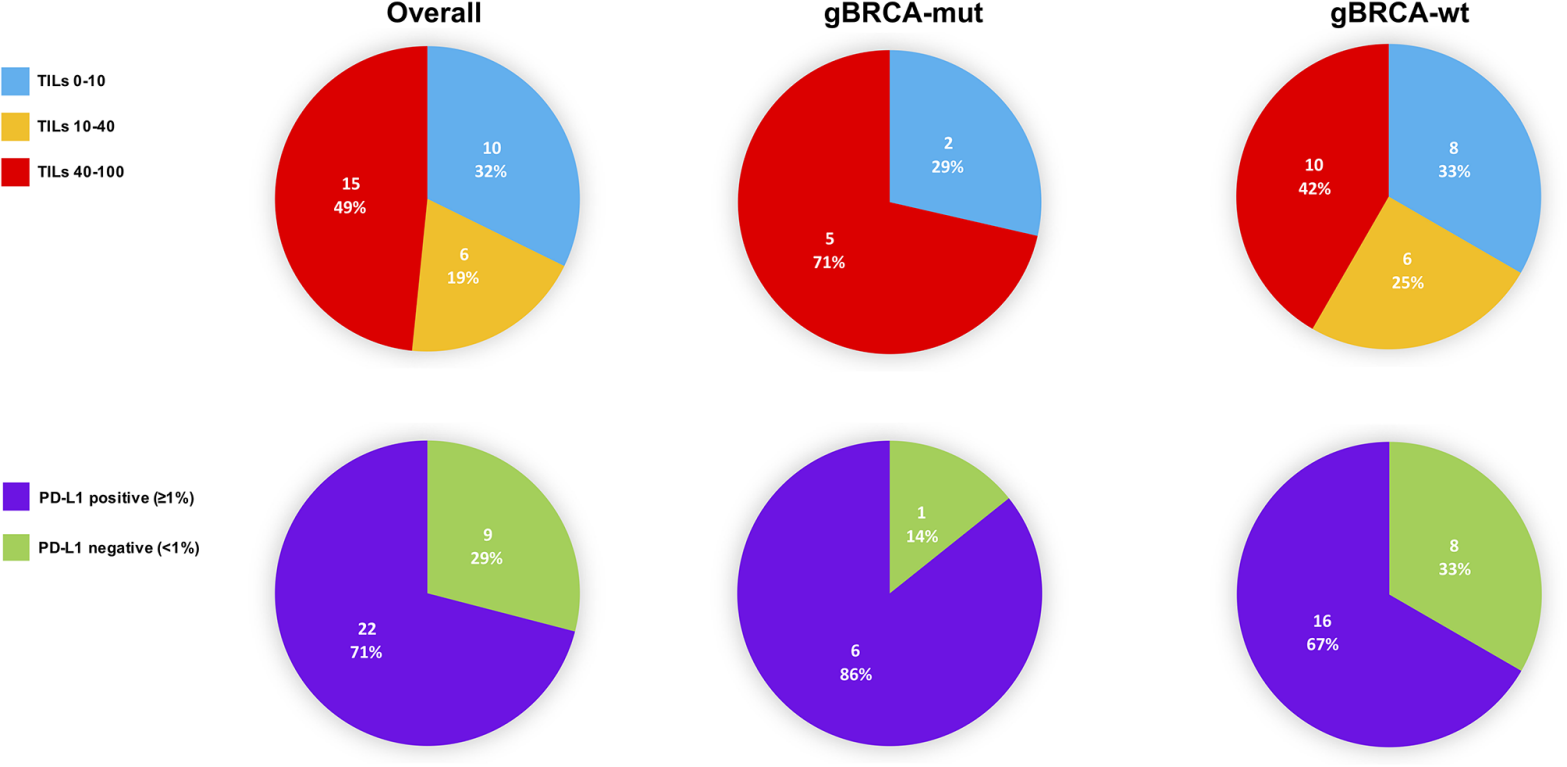
TILs and PD-L1 distribution in the overall population, g*BRCA*-mutant and wild-type tumors at basal assessment. TILs, tumor-infiltrating lymphocytes; g*BRCA*-wt, germinal *BRCA*1/2-wild type triple negative tumors; g*BRCA*-mut, germinal *BRCA*1/2-mutant tumors; PD-L1, PD-L1 in tumor-infiltrating inflammatory cells.

TILs and PD-L1 detection in pathological basal samples was available for 31 patients. The median level of TILs was 40 (min-max: 10 - 90). Median level of expression of PD-L1 was 3% (min-max: 0% - 30%) and 22 (71.0%) patients were considered PD-L1 positive. In g*BRCA*-mutant patients the median value of TILs was 90 (10-90) while in wild-type patients the median was 40 (10-90). More in detail, g*BRCA*-mutant patients showed a numerically higher proportion of tumors with high TILs compared to wild type tumors (5 [71.4%)] *vs.* 10 [41.7%]), whilst the latter presented with a higher proportion of patients with intermediate and low levels of TILs (6 [25.0%] intermediate and 8 [33.3%] low *vs.* 2 [28.6%] low), although non-significant (p=0.388). In mutant patients, a positive PD-L1 was observed in 6 (85.7%) specimens, while 1 (14.3%) was PD-L1 negative. Similarly, 16 (66.7%) wild type tumors were PD-L1 positive, whilst 8 (33.3%) were negative (p=0.639) (**Table 1** and **Figure 2**).

**Table 1:** Demographics at baseline.

Population characteristics	Overall cohort	g*BRCA*-wt	g*BRCA*-mut
	35 (100.0%)	N=27 (77.1%)	N=8 (22.9%)
**Age (years)**			
Mean (SD)	60 ( ± 15)	62 ( ± 15)	54 ( ± 12)
**Germline *BRCA1/2* Mutational Status**			
*BRCA*-mutant	8 (22.9%)	0 (0.0%)	8 (100%)
*BRCA*-wild type	27 (77.1%)	27 (100.0%)	0 (0.0%)
**Breast Cancer Subtype**			
TNBC	33 (94.3%)	27 (100.0%)	6 (75.0%)
HR+/HER2-neg.	2 (5.7%)	0 (0.0%)	2 (25.0%)
HER2+	0 (0%)	0 (0.0%)	0 (0.0%)
**Primary Tumor Dimension (mm)**			
Median (min - max)	39 (18 - 80)	37 (18 -75)	50 (20 - 80)
**TNM Stage**			
I-IIA	0 (0.0%)	0 (0.0%)	0 (0.0%)
IIB-IIIC	35 (100.0%)	27 (100.0%)	8 (100.0%)
IV	0 (0.0%)	0 (0.0%)	0 (0.0%)
**SUV_max_**			
Median (min - max)	9.3 (1.9 - 31.0)	8.7 (1.9 - 31.9)	11.7 (3.8 - 21.9)
**Ki67%**			
Median (min - max)	50% (10% - 90%)	50% (15% – 90%)	63% (10% - 80%)
**TILs**			
Median (min - max)	40 (10 - 90)	40 (10 - 90)	90 (10 - 90)
**PD-L1**			
Negative (<1%)	9 (29.0%)	8 (33.3%)	1 (14.3%)
Positive (≥1%)	22 (71.0%)	16 (66.7%)	6 (85.7%)

Means with standard deviation are reported when the variable distribution was normal according to a Shapiro-Wilk test for normality, otherwise median values with minimum-maximum range are reported; SD, standard deviation; TILs, tumor-infiltrating lymphocytes; SUV, standard uptake volume; PD-L1, PD-L1 in tumor-infiltrating inflammatory cells; gBRCA-wt, germinal BRCA1/2-wild type triple negative tumors; gBRCA-mut, germinal BRCA1/2-mutant tumors.

All patients’ and tumors’ characteristics according to mutational status are reported in **Table 1** and **Supplementary Table 2** for descriptive purposes.

### Clinical and Radiometabolic Response to Olaparib, pCR, and Safety

**Figure 3:**
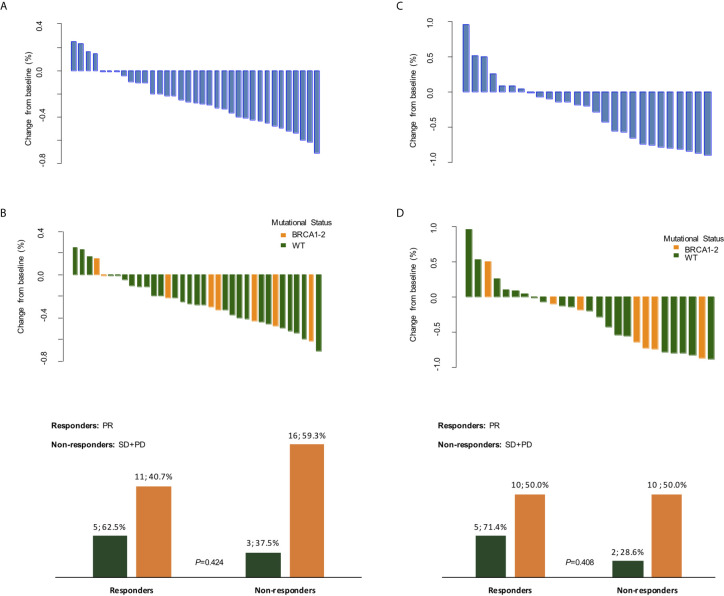
Clinical and radiometabolic responses. Waterfall plots of clinical **(A, B)** and radiometabolic **(C, D)** responses in the overall population and according to BRCA mutational status, along with bar plots **(B, D)** detailing response rates according to BRCA status. PR, partial response; SD, stable disease; PD, progressive disease; WT, wild-type.

Clinical response data were available for all 35 patients after 3 weeks of treatment with olaparib. Of these patients, none achieved a CR, 16 (45.7%) achieved a PR, whilst 17 (48.6%) had SD and 2 (5.7%) experienced PD after 3 weeks of olaparib, before starting neoadjuvant chemotherapy (**Figure 3**). Eight (22.8%) patients refused undergoing PET/CT after olaparib, hence only 27 radiometabolic response evaluations were available. PR was seen in 15 (42.9%) patients, whilst 9 (33.3%) had SD and 3 (23.8%) experienced PD (**Figure 3**). No radiometabolic CR were observed.

Average tumor clinical dimensions decreased from a median of 39mm at diagnosis, to a median of 20mm (p<0.001). Similarly, the average tumor dimension observed at the PET/CT scan slightly decreased from a mean 29.8mm to a mean of 25.9mm (p=0.01). Median SUV_max_ measure decreased from an average value of 9.3 at diagnosis to 4.8 at the end of olaparib treatment, as well (p=0.004) (**Table 2**).

**Table 2:** Clinical and radiometabolic responses, pathologic and immunologic changes induced in the overall population by olaparib.

Variables	Pre-olaparib	Post-olaparib	P*
**Clinical Dimension (mm)**			
Median (Min - max)	39 (18 - 80)	20 (0 - 80)	*<0.001*
Δ **Clinical Dimension**			
Mean (SD)	**-**	-25.3 ( ± 24.5)	–
**Clinical Response After Olaparib**			
Complete Response	–	0 (0.0%)	–
Partial Response	–	16 (45.7%)	
Stable Disease	–	17 (48.6%)	
Progressive Disease	–	2 (5.7%)	
**PET/CT dimensions (mm)**			
Mean (SD)	29.8 ( ± 17.4)	25.9 ( ± 17.5)	*0.01*
**SUV_max_**			
Median (Min - max)	9.3 (1.9 - 31.0)	4.8 (1.0 - 23.8)	*0.004*
Δ **SUV_max_**			
Mean (SD)	–	-27.1 ( ± 49.3)	–
**Radiometabolic Response After Olaparib**			
Complete Response	–	0 (0.0%)	–
Partial Response	–	15 (42.9%)	
Stable Disease	–	9 (33.3%)	
Progression Disease	–	3 (23.8%)	
**Pathologic Response After Surgery**			
pCR	–	8 (22.9%)	–
No pCR	–	22 (62.9%)	
Not Available	–	5 (14.2%)	
**Ki67%**			
Median (min - max)	50.0 (10 - 90)	62.5 (0 - 90)	0.67
**TILs**			
Median (min - max)	40 (10 - 90)	40 (0 - 90)	0.77

Means with standard deviation are reported when the variable’s distribution was normal according to a Shapiro-Wilk test for normality, otherwise median values with minimum-maximum range are reported; Δ, Delta; Δ formula, (Post-therapy value - Baseline Value)/Baseline Value; SD, standard deviation; TILs, tumor-infiltrating lymphocytes; SUV, standard uptake volume; min, minimum; max, maximum. *, exploratory comparisons; pCR, pathologic complete response.

When we explored the clinical and radiometabolic response rates in g*BRCA*-mutant *vs.* wild-type tumors, the proportion of responders was numerically higher for the first compared to the latter group in both cases, albeit non statistically significant (62.5% *vs.* 40.7%, p=0.42 and 71.4% *vs.* 50.0%, p=0.41) (**Figure 3** and **Supplementary table 3**). Both clinical dimension Δ in g*BRCA*-mutant tumors compared to wild-type TNBC (-27.8% [ ± 25.0%] *vs.* -24.5% [ ± 24.7%]), and SUV_max_ variations (-40.1% [ ± 49.4%] *vs.* -22.6% [ ± 50.0%]) were more accentuated for the first group, despite the lack of a statistically significant difference (p=0.74 and p=0.43, respectively).

A precise evaluation of the pCR rates was out of the scope of this study. However, at the end of the trial, pathology data after surgery were available for 30 (85.7%) patients. Seven out of 30 (23.3%) achieved a pCR (**Table 2**). More in detail, 2/8 (25.0%) g*BRCA*-mutant patients showed a pCR compared to 5/27 (18.5%) non-mutant patients. This numerical difference did not translate into a statistically significant different distribution of pCR between mutant and non-mutant patients (p=0.588). All patients had been treated with neoadjuvant chemotherapy following the 3 weeks of olaparib (26). The regimen administered was the standard combination of three-weekly epirubicin (90mg/m^2^) and cyclophosphamide (600mg/m^2^) for 4 cycles, followed by 12 cycles of weekly paclitaxel (80mg/m^2^).

Olaparib toxicity profile in BC has already been well established (8). No unexpected toxicities were observed. Ten out of 35 patients (28.57%) developed grade (G)1 or 2 gastrointestinal adverse reactions during treatment. The most common was nausea (4 cases, 3 of G1 and 1 of G2), followed by G1 gastric pain and constipation. No ≥G3 adverse events requiring dose reduction or treatment interruption were recorded.

### Pathologic Biomarkers’ Variations Induced by Olaparib

No significant change in median Ki67 percentage was observed in the overall population after olaparib (p=0.67), along with no significant Ki67 variation induced by olaparib in g*BRCA-*wild-type *vs.* mutant cases (p=0.85) (**Supplementary Tables 2 and 3**).

Similarly, TILs did not differ significantly from basal after olaparib (p=0.77) and no significant variation after olaparib was also observed when comparing mutant *vs.* wild-type tumors (p=0.26) (**Supplementary Tables 2 and 3**).

PD-L1 expression was evaluable only in 20 post-olaparib specimens and biomarker variation pre/post olaparib was assessable in 19 paired samples. PD-L1 median levels did not differ before and after treatment (3% [min – max: 0% - 30%] *vs.* 3% [min – max: 1% - 23%], p=0.17), with PD-L1 positive immune cells (18 [90.0%]) still prevailing over PD-L1 negative (2 [10.0%]) after olaparib (p=0.13), with a slight numerical increase in the overall number of PD-L1 positive cases (p=0.134).

### Clinico-Pathological and Radiometabolic Biomarkers According to Treatment Response

When we divided patients according to clinical response in responders *vs.* non-responders, the Δ of clinical dimension and SUV_max_ observed in responders *vs.* non-responders differed significantly (-45.9 [SD: ± 0.12] *vs.* -0.08 [SD: ± 0.17], p<0.001 and -0.5 [SD: ± 0.4] *vs.* -8.9 [SD: ± 0.5], p=0.03, respectively). Conversely, no significant differences in the pre/post olaparib variation (Δ) of Ki67 and TILs were observed between the two groups (p=0.20 and p=0.75) (**Supplementary Table 4**).

Similarly, when we considered radiometabolic responders *vs.* non-responders, the Δ for clinical dimension and SUV_max_ differed significantly (-0.34 [SD: ± 0.21] *vs.* -0.10 [SD: ± 0.27], p=0.01 and -0.62 [SD: ± 0.25] *vs.* 0.17 [SD: ± 0.34], p<0.001, respectively) but no differences in the Ki67 and TILs’ Δ were observed (p=0.70 and p=0.92) (**Supplementary Table 4**).

Despite not showing changes in TILs dynamics after olaparib therapy, clinical and radiometabolic responders showed numerically higher levels of basal TILs (90 [min – max: 10 - 90] in both cases), in comparison to clinical and radiometabolic non-responders (40 [min – max: 10 - 90] and 25 [min-max: 10-90], respectively), albeit not statistically significant (p=0.13 and p=0.07, respectively). Conversely, similar basal values of T-regs/T-eff count and % were observed between clinical and radiometabolic responders and non-responders (**Supplementary Table 5**).

Clinical responders, compared to non-responders, showed a numerically higher proportion of PD-L1 positive cases (13 [86.7%] *vs*. 9 [56.3%]), though non-significant (p=0.11). The finding was similar when observing radiometabolic responders compared to non-responders (12 [80.0%] *vs*. 7 [63.6%], p=0.41) (**Supplementary Table 5**).

PD-L1 expression did not change in both clinical and radiometabolic responders, while a similar proportion of clinical (4/13 [30.8%]) and radiometabolic (3/8 [37.5%]) non-responders experienced a change in PD-L1 expression. More specifically, in both cases, PD-L1 negative tumors became positive after treatment (4/5 [80.0%] and 3/3 [100.0%] for clinical and radiometabolic non-responders, respectively; p=0.13 and p=0.25), while no other changes were observed (**Table 3**).

**Table 3:** PD-L1 status changes in olaparib responders and non-responders.

PD-L1 Status Pre/Post Olaparib	Clinical Responses			Metabolic Responses		
	Responders	Non-Responders	*P*	Responders	Non-Responders	*P*
*Positive to Negative*	0	0	0.13	0	0	0.25
*Negative to Positive*	0	4		0	3	
*Negative to Negative*	1	1		1	0	
*Positive to Positive*	5	8		7	5	

An assessment of PD-L1 expression status according to *BRCA* mutational status could not be performed, since only 2 patients with mutant tumors had PD-L1 data available.

### Olaparib Effect on Immune Circulating Cells

**Figure 4:**
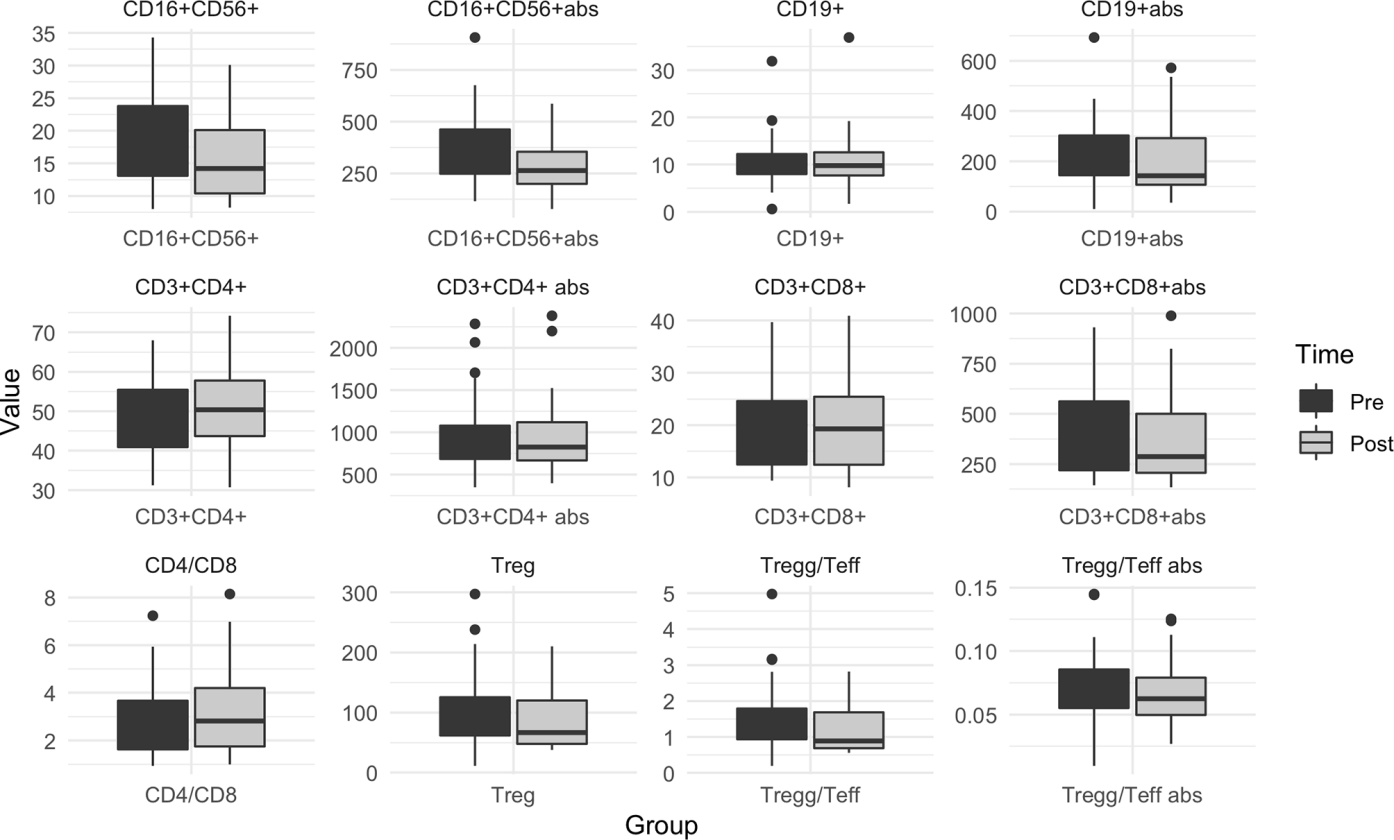
Box plot for pre/post olaparib circulating immune cells levels in the overall population. Pre: before olaparib; Post: after 3 weeks of olaparib; Abs, absolute count. When abs not specified, the graphic is referred to %. Treg, Regulatory T lymphocytes; Teff, Effectors T Lymphocytes.

Circulating lymphocytes subpopulations were evaluated before and after olaparib administration. No differences were observed in overall circulating T helper (p=0.76), T-regs (p=0.12) and T suppressor (p=0.10) absolute counts, although a small and marginally significant reduction in T-regs/T-eff count and % were observed (p=0.05 and p=0.04, respectively) (**Figure 4** and **Supplementary Table 6**). At the same time a significant reduction in the absolute count of B lymphocytes (from average 226.27 cells/Ul [SD: ± 139.01] to 195.03 [SD: ± 128.39], p=0.003) and NK lymphocytes (from average 366.88 [SD: ± 182.46] to 282.18 [SD: ± 111.84], p=0.003) was observed, which translated also in an average increase of the T helpers rate (from 47.21% [SD: ± 9.49%] to 51.00% [SD: ± 9.81%], p<0.001) and CD4/CD8 ratio (from 2.90 [SD: ± 1.61] to 3.26 [SD: ± 1.89], p=0.02), accompanied by a significant decrease in the mean NK lymphocytes rate (18.73 [SD: ± 7.84] to 16.02 [SD: ± 6.44], p=0.004) (**Figure 4** and **Supplementary Table 6**).

No significant differences in the variations of immune circulating cells was observed according to g*BRCA* mutational status (**Supplementary Table 3**).

When comparing all pre/post-olaparib variations in circulating immune biomarkers according to clinical and radiometabolic response status, no significant differences were observed (**Supplementary Table 4**).

Finally, we investigated the correlation between basal T-regs and CD4+/CD8+ ratio with PD-L1 status (positive *vs.* negative) but did not find any (data not shown).

### Gene Mutations and Responses to Olaparib

**Figure 5:**
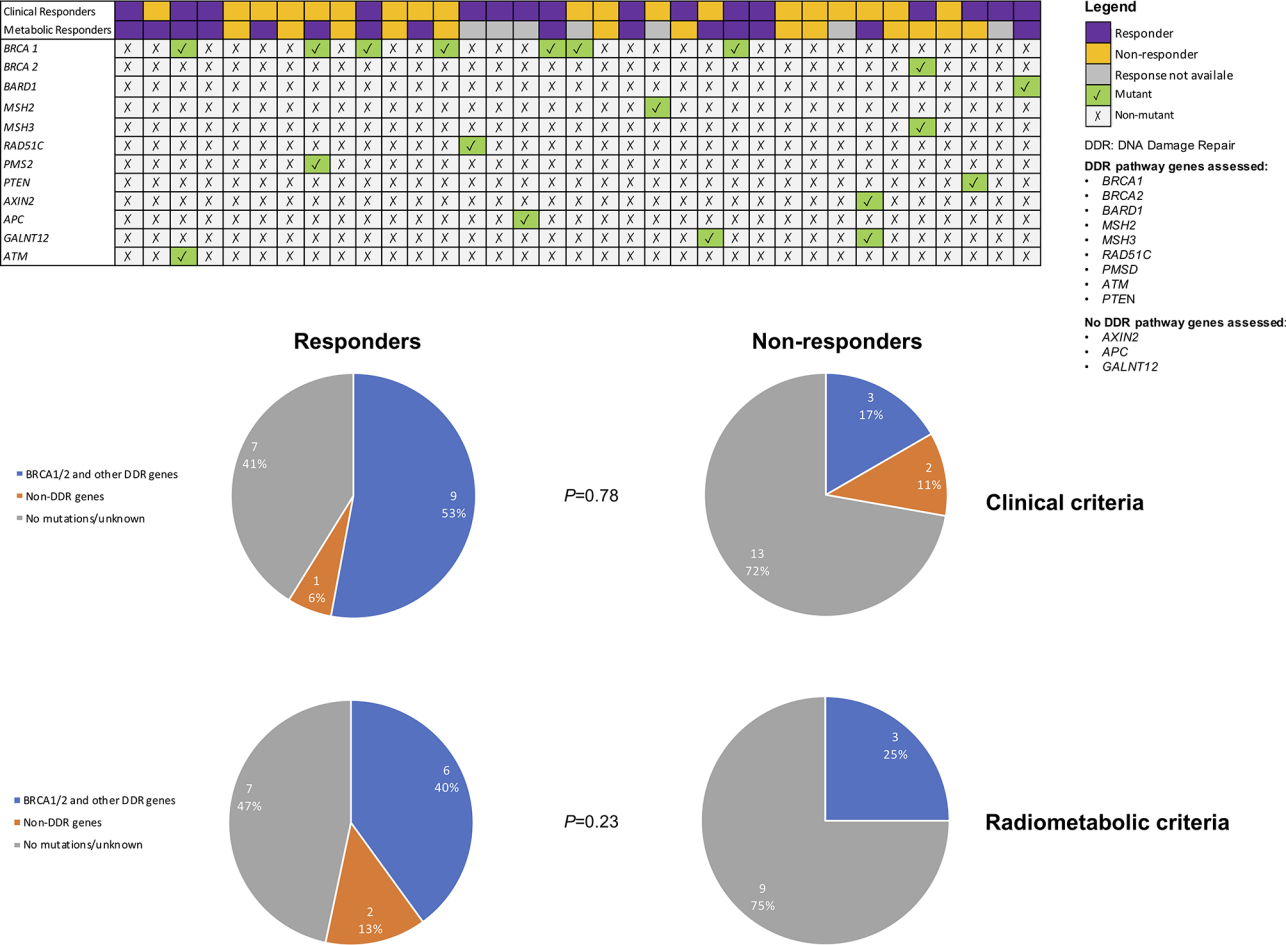
Mutational status according to clinical and radiometabolic response status. Only mutant genes are reported.

The distribution of germline mutations detected in clinical and radiometabolic responders and non-responders was evaluated and no statistically significant differences were observed in both cases (p=0.78 and p=0.23, respectively) (**Figure 5**). In any case, both clinical and radiometabolic responders showed a numerically higher rate of patients detected with mutations in genes involved in DDR pathways, compared to non-responders (52.9% and 40.0% *vs.* 16.7% and 25.0%, respectively). Conversely, in clinical and radiometabolic non-responders prevailed a numerically higher rate of non-mutant patients, compared to responders (72.2% and 75.0% *vs.* 41.2% and 46.7%, respectively). Patients with mutations in genes not involved in DDR were not present among radiometabolic non-responders, but were slightly more frequent in clinical non-responders *vs.* responders (11.1% *vs.* 5.9%, respectively). When considering only g*BRCA* mutations, 4 (50.0%) patients were both clinical and radiometabolic responders, while 1 (12.5%) patient achieved only a clinical response and another 1 (12.5%) only achieved a radiometabolic response (**Figure 5**).

## Discussion

OLTRE was a WoO trial that aimed to assess primarily the biologic, immunologic and genetic changes that olaparib might induce in g*BRCA*-wild type TNBC, with a small cohort of HER2-negative g*BRCA*-mutant tumors as proof-of-concept. It represented also an opportunity to preliminarily assess the potential role of olaparib as an induction treatment before neoadjuvant chemotherapy.

Although the trial was not specifically powered to assess short-course olaparib activity, it is interesting to note that a significant reduction in tumor clinical dimension and SUV_max_ were observed, with significant differences in the Δ between clinical and radiometabolic responders *vs.* non-responders. Notably, a more pronounced Δ was observed in g*BRCA*-mutant tumors, compared to g*BRCA*-wild type TNBC, with respect to both clinical and radiometabolic responses, but the difference was not significant and response rates did not differ significantly between the two study arms. These results suggest that olaparib might be effective also in g*BRCA*-wild type TNBC, similarly to what observed in the PETREMAC phase II neoadjuvant trial, where a 56.3% of objective responses were observed in the overall unselected TNBC population (27). In fact, in our study, 40.7% of g*BRCA*-wild type TNBC and 62.5% of mutant tumors achieved a clinical response, with even higher rates of radiometabolic responders (71.4% and 50.0%). Importantly, while within the PETREMAC trial olaparib was administered for at least 10 weeks, without chemotherapy, our study only focused on a brief 3-weeks olaparib course. These results, taken together, suggest that olaparib might be effective in unselected TNBC, and that responses might occur quickly, already within the first 3 weeks of treatment. However, Ki67 levels did not appear to be affected by olaparib short course treatment, since no significant modifications were observed. A reduction in Ki67% levels usually suggest an antiproliferative effect (28), and several neoadjuvant trials in breast cancer have provided evidence that biomarkers representing proliferation-related processes can provide additional prognostic information over pCR (28–31). In this perspective we could not observe a direct antiproliferative effect of olaparib. It is possible that this is due to the fact that olaparib is involved in inducing tumor cell death, without directly interfering with the cell cycle (32), differently from other target agents such as CDK4/6-inhibitors (28). This is coherent with the significant tumor dimension reduction, accompanied by a reduced glucose uptake from cancer cells, as observed at the FDG^18^-PET/CT.

There is growing evidence highlighting the high rate of DDR response deficiency across triple negative tumors and decreased expression of DNA repair genes (6), which implies the possibility that PARP inhibitors might be effective also in in *BRCA*-wild type TNBC. This concept is also reinforced by growing evidence of DNA damaging agents, such as platinum salts, being particularly effective in this breast cancer subset (33). In our study, the activity of olaparib in g*BRCA*-wild type TNBC might be, at least in part, the result of the presence of several mutations in genes involved in DDR pathways, directly (e.g. *RAD51C, BARD1, PMS2*) (34, 35) or indirectly (e.g. *PTEN*) *(* 36). In fact, we observed that 53% of clinical responders and 40% of radiometabolic responders carried mutations in *BRCA1/2* or other DDR-involved genes. At the same time, 41% of clinical and 47% of radiometabolic responders, were wild-type TNBC, at least with respect to the mutations assessed with our gene panel. This evidence further supports the idea that olaparib might be active also in unselected TNBC, possibly due to a constitutive downregulation of DNA repair genes (6), or the presence of not yet defined genetic variants, not captured by our panel. Less clear is the role of the genetic mutations in non-DDR genes such as *GALNT12*, *AXIN2* and *APC* observed in 3 patients. In fact, 2 patients harboring germline variants of *GALNT12* and *AXIN2* did not respond clinically, but showed a radiometabolic response. The first is a gene codifying for an enzyme catalyzing the initial reaction in O-linked oligosaccharide biosynthesis; the latter is a gene that is likely to play an important role in the beta-catenin-Wnt pathway (37–39). These genes, when impaired, have been associated to increased risk of colorectal cancer (both) and breast cancer (*AXIN2*) (37–39). The one patient with germline APC mutation, conversely, experienced a clinical response. APC is usually associated with the development of colorectal cancer or, when germline mutant, with the familial adenomatous polyposis (FAP) (40). It is involved in the regulation of the Wnt pathway, cell migration and adhesion processes, as well as apoptosis (40).

There are evidences supporting a potential interplay between immune system and PARP inhibitors (41). The vast majority of our patients carried TNBC, irrespective of *BRCA* mutational status. Therefore, as expected, median TILs levels were relatively high (42). Coherently with what elsewhere reported, g*BRCA*-mutant tumors seemed to be more enriched in TILs than wild-type TNBC (43). In the PETREMAC trial, TNBC responding to olaparib were characterized by high TILs and PD-L1 expression levels (27). In our study, both clinical and radiometabolic responders showed higher numerical proportion of TILs, compared to non-responders. Additionally, olaparib seemed not to have an influence on TILs levels, nor in the overall population, neither according to treatment response or *BRCA* mutational status. Therefore, basal TILs might be a potential biomarker of response to olaparib, especially in g*BRCA*-wild type TNBC, where olaparib efficacy is more questionable if mutations in DDR genes are not present or not assessable. Conversely, there is no sign of a clinical utility of TILs dynamics with respect to olaparib in non-metastatic TNBC.

Interestingly, clinical and radiometabolic responders presented with numerically higher levels of PD-L1 positive cases compared to non-responders. It is important to highlight that PD-L1 positivity was assessed through the standard diagnostic methodology currently adopted in phase III trials of immune checkpoint inhibitors in TNBC, which is also the only FDA-approved for theranostics of the anti-PD-L1 atezolizumab in metastatic TNBC (i.e. Ventana SP142). At the same time, this test does not take into account PD-L1 expression on tumor cells, which has been recently suggested to provide additional prediction of benefit from immunotherapy in TNBC in the Keynote trials, when combined to PD-L1 expression detection on immune cells (44–46). Moreover, the SP142 assay has been demonstrated to provide lower sensitivity than other PD-L1 detection assays (47).

Considering that PD-L1 has been demonstrated to predict response to immune checkpoint inhibitors in TNBC, and that, along with TILs and *BRCA* mutational status, seems to be correlated to better response to olaparib in this and other studies (27), further trials of experimental combinations of olaparib and anti-PD-L1 (e.g. atezolizumab or pembrolizumab) prior to standard CT are justified (48, 49). Additionally, efforts towards the definition of a more standardized and comprehensive approach for the detection of PD-L1 on both tumor cells and immune cells should be a priority for the scientific community, in order to provide the highest possible benefit from immune-checkpoint inhibitors of the PD1/PD-L1 axis to the broadest possible patients population.

Considering also that TNBC with high TILs have shown a relatively good prognosis in early stage, independently from chemotherapy administration, a proportion of selected TNBC (e.g. g*BRCA*-mutant, with high basal TILs and PD-L1 positive) might be candidate to chemotherapy-sparing neoadjuvant trials. In these cases, the decision to whether administer or not subsequent adjuvant chemotherapy might then be based on post-surgery pCR status, given the established prognostic role of pCR in TNBC (50).

Preclinical studies have shown the existence of a cross-talk between PARP inhibition and tumor-associated immunosuppression, with reported increase in PD-L1 expression after therapy (51, 52). Notably, we observed a similar finding, although a formal statistical comparison was impaired by the low patients’ number. These data collectively suggest that anti-PD-L1 immune-checkpoint inhibitors and olaparib might be good therapeutic partners, sequentially or in combination, since a better response to the former might be favored by the induction of an increased PD-L1 expression by the latter. However, the relationship between PD-L1 and PARP inhibition warrants further investigation in *ad hoc* studies.

One of the common adverse events observed with olaparib is leukopenia, with reductions in both neutrophile and lymphocyte counts. Therefore, it is not surprising that lymphocytes reduction was observed within our study. When analyzing the lymphocytes subpopulations, we observed that olaparib mostly reduced B and NK cells absolute count, while slightly increasing T-regs, a subpopulation of CD4+ T lymphocytes, so determining an increase in CD4+/CD8+ ratio. The ratio between T-regs and T-effectors seemed also to be slightly affected by olaparib, with a limited reduction. These findings are in line with preclinical evidence showing B lymphocytopenia in PARP1/2-deficient mice and, at the same time, in discordance with other studies showing also a peripheral T cell reduction in case of PARP deficiency (41). In any case, no significant differences was observed according to treatment response status, nor numerically neither statistically. This finding suggests that the dynamics of lymphocytes subpopulation, although affected by olaparib, cannot help predict treatment response, at least not as an early response biomarker. Nevertheless, a broad spectrum of immunomodulatory effects has been observed preclinically in PARP-deficient mice and the interplay between PARP enzymes and immune cell function is complex and yet to be clearly elucidated (41). Although we did not observe a clear relation between lymphocytes dynamics and response to olaparib, this does not exclude a functional effect, as suggested by important preclinical evidence (41). Further studies are needed, in this perspective.

With respect to the safety profile, 3-weeks olaparib was well tolerated without unexpected adverse events and no G3-5 toxicities. Importantly, within the brief 21-days period of olaparib administration, the observed leucopenia did not translate into clinically relevant side effects, nor required treatment suspension and/or dose modifications.

Surprisingly, the assessment of pCR rates yielded poor results, compared to what observed for TNBC and *BRCA*-mutant tumors in the literature (33, 50). This could be partly explained by the pathology reports missing rate (14%), however without precise data on schedules, toxicities and treatment discontinuations, it is difficult to draw any conclusion. Nevertheless, this was out of the scope of this study.

Several limitations need to be acknowledged. This study was single-center, non-randomized and open-label, with a limited number of patients. Furthermore, being a window-of-opportunity trial, it was not specifically powered to assess olaparib activity and efficacy in the neoadjuvant setting, and all statistical analyses have to be considered as exploratory. Nevertheless, it was an opportunity to preliminarily investigate olaparib activity in *BRCA*-wild type TNBC and to detect potentially relevant biologic changes that might merit further investigation or might be useful in the design of novel clinical trials with PARP-inhibitors and immune-checkpoint inhibitors. Reassuringly, a recently published phase II neoadjuvant trial with olaparib in about 200 unselected TNBC patients, reported some results in line with what observed within the OLTRE (27). More specifically, in both studies, high basal TILs and PD-L1 seemed to correlate with higher response rates to olaparib. Additionally, a significant percentage of responders in both studies appeared to carry germinal deficiencies in genes involved in DNA damage repair, either through homologous recombination (both studies), or mismatch repair and other genes (only in our trial) (27).

In conclusion, study results, along with current evidence, suggest that: 1) unselected TNBC or, at least, TNBC profiled with gene panels assessing DDR-pathways deficiencies beyond the mere g*BRCA1/2-*mutations, are a key candidate target population for olaparib (and ideally other PARP-inhibitors) studies; 2) future trials should combine basal TILs, PD-L1 and *BRCA* mutational status to more adequately identify candidates for escalated or de-escalated neoadjuvant approaches in TNBC patients with PARP-inhibitors; 3) future combination or sequential trials of PARP-inhibitors and immune-checkpoint inhibitors relying on PD-L1 positivity to be effective (e.g. anti-PD-L1) are warranted, since PARP-inhibitors might increase PD-L1 positivity rates; 4) the interplay between PARP inhibition and immune system needs to be more precisely assessed in both preclinical and translational studies. Gene and protein expression analysis from pre/post olaparib samples, as well as further correlative secondary biomarker studies from the OLTRE are ongoing and will hopefully help to shed a light on some of these aspects.

## Data Availability Statement

The original contributions presented in the study are included in the article/**Supplementary Material**. Further inquiries can be directed to the corresponding authors.

## Ethics Statement

The studies involving human participants were reviewed and approved by Ethical Committee of the ASST of Cremona Hospital. The patients/participants provided their written informed consent to participate in this study.

## Author Contributions

DG conceived the study. All authors, except for FS and FG, collected the data and participated in study procedures, along with study nurses, clinical research coordinators and other study-center staff. FG performed the statistical analyses. FS, SC and DG interpreted the results and wrote the first manuscript draft. All authors contributed to the article and approved the submitted version.

## Funding

The study has been conducted with Astra-Zeneca contribution. The funder had no role in the design of the study; in the collection, analyses, or interpretation of data; in the writing of the manuscript, or in the decision to publish the results. This research was also supported by Mednote, spin-off - University of Trieste - Mozart Program.

## Disclaimer

Any views, opinions, findings, conclusions, or recommendations expressed in this material are those solely of the author(s) and do not necessarily reflect those of ESMO.

## Conflict of Interest

EM, AB, LX, EZ and DC were employed by Personal Genomics Ltd. GW and ML are employed at Oncologica UK Ltd., which has received project funding from AstraZeneca outside of the submitted work. DG has declared consulting fees from Novartis, Lilly and Pfizer, research funding from LILT, Novartis, Astra-Zeneca and University of Trieste outside of the submitted work. IP has declared consulting fees from Roche, Novartis, Lilly, Pfizer, Astra-Zeneca, Pierre Fabre and Ipsen outside of the submitted work. GS has declared Grant/Research Support from MSD Italia S.r.l., consulting role for TESARO Bio Italy S.r.l. Johnson & Johnson and Clovis Oncology Italy S.r.l., outside of the submitted work.

The remaining authors declare that the research was conducted in the absence of any commercial or financial relationships that could be construed as a potential conflict of interest.
