# Supplementary material for: Clinical, Radiometabolic and Immunologic Effects of Olaparib in Locally Advanced Triple Negative Breast Cancer: The OLTRE Window of Opportunity Trial
Source: Front Oncol. 2021 Jun 28;11:686776. doi: 10.3389/fonc.2021.686776 (PMC8273330; doi:10.3389/fonc.2021.686776)
Supplement: Supplementary file 1 [file DataSheet_1.pdf]

Supplementary Materials

Supplementary Methods

PD-L1 testing details

VENTANA SP142 CDx assay consists of the rabbit monoclonal anti-PD-L1 SP142 clone, the OptiView DAB IHC detection kit, the Opti-View Amplification Kit stained on the VENTANA BenchMark ULTRA instrument using the staining protocol provided by the package insert and interpreted with the guidelines of the VENTANA interpretation guide. All cases have an accompanying hematoxylin & eosin (H&E)-stained patient slide, negative reagent control-stained patient slide, with an on-slide tonsil control, and a VENTANA PD-L1 SP142-stained patient slide with an on-slide tonsil control. PD-L1 IHC slides were interpreted using the tumor-infiltrating immune cell (IC) percentage scoring method, where IC% = proportion of tumor area that is occupied by PD-L1 staining IC of any intensity. Each case was interpreted by two independent pathologists who were trained specifically in the SP142 PD-L1 TNBC CDx assay interpretation, and borderline cases (close to 1% IC positive) were reviewed by the two pathologists to arrive at a consensus score.

Flow cytometry analysis

The lymphocytes subpopulations (B, NK, T with CD4 and CD8 subpopulation) were assessed with BD Multitest 6-Color TBNK kit (Becton Dickinson™). The kit contains FITC-labelled CD3 (SK7clone), PE-labelled CD16 (B73.1 clone) and CD56 (NCAM 16.2 clone), CD45 (2D1 clone) conjugated with the fluorochromes PerCP-Cy5.5, CD4 (SK3 clone) conjugated with PE-Cy7, CD19 (SJ2SC1 clone) conjugated with APC and CD8 (SK1 clone) conjugated with APC-Cy7. The BD FACSCanto clinical software was employed to carry out the analysis. Leucocytes were identified by CD45 expression and SSC/FCS morphological parameters. T lymphocytes were sorted by CD3 expression and then split into CD4 and CD8 populations. CD3 negative cells were split into B lymphocyte (expressing CD19) and NK cells (CD16 and CD56 positive). Subpopulations' absolute count was done by the "trucount tube" (BD™) containing a known number of beads. The T-reg cells (CD4 positive, bright CD25 positive and CD127 negative) were sorted using single Becton Dickinson monoclonal antibodies: CD3 (SK7 clone) conjugated with the fluorochromes FITC, CD4 FITC, CD25 (2A3 clone) conjugated with PE, CD4 (SK3 clone) conjugated with PerCP-Cy5.5 and CD127 (HIL-7R-M21 clone) conjugated with V450, CD45 (HI30 clone) conjugated with V500.

Mutational status analysis

The Kapa Hyperplus kit consists of enzymatic, end repair, a-tailing and adaptor ligation steps followed by purification with Agentcourt AMPure XP reagent (Beckman Coulter, Beverly, MA, USA) and PCR amplification. Up to 12 libraries were pooled and hybridized with biotinylated probes of SeqCap EZ Choise kit, followed by wash steps and PCR amplification according to manufacturer's instructions. Quantity and quality of prepared libraries were assessed by Qubit® 3.0 fluorometer (Thermofisher Scientific) and Labchip electrophoresis (Perkin Elmer) respectively, as previously described (Simbolo M et al. PloS One 2013; 8(6):e62692). Subsequently, libraries were mixed in an equimolar ratio and sequencing was performed using the Illumina Miseq (Illumina).

Raw reads were filtered using fastp (version 0.20.1); the filtered reads were mapped on the HG38 reference genome using BWA-mem (version 0.7.17-r1188); PCR duplicates removal and base recalibration was performed with GATK (version 4.1); variant were called using HaplotypeCaller (GATK version 4.1) and hard-filtered with VariantFiltration (GATK version 4.1). Finally, the VCF files were annotated with VarSeq by GoldenHelix.

Genetic variants detected in the analyzed genes were validated by Sanger sequencing using a BigDye Therminator 3.1 Cycle Sequencing Kit (Life Technologies, Carlsbad, CA, USA) and readthrough the 3130xl Genetic Analyzer (Applied Biosystems, Foster City, CA, USA), according to manufacturer’s protocols.

Supplementary tables

Supplementary table 1. Germline mutations found and their frequency in the study population

Mutation	Overall Cohort (N=35)
BRCA 1	7 (20%)
BRCA 2	1 (2.8%)
BARD1	1 (2.8%)
MSH2	1 (2.8%)
MSH3	1 (2.8%)
RAD51C	1 (2.8%)
PMS2	1 (2.8%)
PTEN	1 (2.8%)
AXIN2	1 (2.8%)
APC	1 (2.8%)
GALNT12	2 (5.6%)

Supplementary table 2. Circulating immune cells at baseline

Population characteristics	Overall cohort	g*BRCA*-wt	g*BRCA*-mut
	35 (100.0%)	N=27 (77.1%)	N=8 (22.9%)
**Circulating CD3+CD4+ T Lymphocytes %**			
Mean (SD) or Median (min - max)	47.21 (±9.49)	47.3 (31.2 - 68.0)	44.3 (31.4 - 62.8)
**Circulating CD3+CD4+ T Lymphocytes Count (cells/Ul)**			
Mean (SD) or Median (min - max)	961 (±451)	897 (347 - 2285)	687 (412 - 1310)
**Circulating T-reg (cells/Ul)**			
Median (min - max)	80 (12 - 297)	79 (12 - 297)	108 (32 - 139)
**Circulating CD3+CD8+ T Lymphocytes %**			
Median (min - max)	19.0 (9.4 - 7.0)	20.1 (9.4 - 39.7)	19.0 (10.5 - 34.0)
**Circulating CD3+CD8+ T Lymphocytes Count (cells/Ul)**			
Median (SD)	361 (144 - 932)	365 (144 - 932)	277 (169 - 584)
**CD4+/CD8+ Ratio**			
Mean (SD) or Median (min - max)	2.90 (±1.61)	2.7 (0.9 - 7.2)	2.4 (1.2. -5.9)
**T-regs/T-eff ratio**			
Mean (SD)	0.07 (±0.03)	0.07 (±0.03)	0.08 (±0.03)
**T-regs/T-eff ratio%**			
Median (min - max)	1.17 (0.20 - 4.97)	1.13 (0.20 - 4.97)	1.47 (1.02 - 3.15)
**Circulating CD19+ B Lymphocytes %**			
Mean (SD) or Median (min - max)	11.11 (±5.34)	10.8 (4.1 - 19.3)	12.1(0.6 - 31.9)
**Circulating CD19+ B Lymphocytes Count (cells/Ul)**			
Mean (SD) or Median (min - max)	226 (±139)	180 (64 - 449)	231 (10 - 693)
**Circulating CD16+CD56+ NK Lymphocytes %**			
Mean (SD) or Median (min - max)	18.73 (±7.84)	15.6 (9.1 - 34.3)	13.1 (8.0 - 27.3)
**Circulating CD16+CD56+ NK Lymphocytes Count (cells/Ul)**			
Mean (SD) or Median (min - max)	367 (±182)	344 (152 - 906)	252 (116 - 462)

Legend and captions. Means with standard deviation are reported when the variable distribution was normal according to a Shapiro-Wilk test for normality, otherwise median values with minimum-maximum range are reported; SD: standard deviation; TILs: tumor-infiltrating lymphocytes; SUV: standard uptake volume; PD-L1 TC: PD-L1 in tumor cells; PD-L1 IC: PD-L1 in tumor-infiltrating inflammatory cells; T-regs/T-eff ratio: T.regs count or %/(CD3+CD4+ count or % + CD3+CD8+ count or %); g*BRCA*-wt: germinal *BRCA*1/2-wild type triple negative tumors; g*BRCA*-mut: germinal *BRCA*1/2-mutant tumors.

Supplementary table 3. Response patterns and changes in pathologic and immune circulating markers according to g*BRCA* status

Δ Pre/Post Treatment and Response Rates	g*BRCA*-wt	g*BRCA*-mut	***P****
	(n=27)	(n=8)	
**Clinical Response After Olaparib**			

Complete Response	0 (0.0%)	0 (0.0%)	
Partial Response	11 (40.7%)	5 (62.5%)	0.42
Stable Disease/Progression Disease	16 (59.3%)	3 (37.5%)	
**Δ Clinical Dimension**			
Mean (SD)	-24.5% (±24.7%)	-27.8% (±25.0%)	0.74
**Radiometabolic Response After Olaparib**			
Complete Response	0 (0.0%)	0 (0.0%)	
Partial Response	10 (50.0%)	5 (71.4%)	0.41
Stable Disease/Progression Disease	10 (50.0%)	2 (28.6%)	
**Δ SUV (%)**			
Mean (SD)	-22.6% (±50.0%)	-40.1% (±49.4%)	0.43
**Δ Ki67 %**			
Median (Min-max)	0.0 (-0.2; 1.5)	0.0 (-0.1; 1.6)	0.85
**Δ TILs**			
Median (Min-max)	0.0 (-1.0; 8.0)	0.0 (-1.0; 0.0)	0.26
**Δ Circulating CD3+CD4+ T Lymphocytes %**			
Median (Min-max)	0.07 (-0.05; 0.31)	0.03 (-0.05; 0.15)	0.72
**Δ Circulating CD3+CD4+ T Lymphocytes Count**			
Median (Min-max)	-0.03 (0.34; 0.70)	-0.07 (-0.18; 0.23)	0.8
**Δ Circulating T-regs**			
Median (Min-max)	-0.16 (-0.78; 2.91)	-0.25 (-0.5; 0.3)	0.33
**Δ Circulating CD3+CD8+ T Lymphocytes %**			
Median (Min-max)	0.01 (-0.15; 0.48)	-0.01 (-0.13; 0.08)	0.55
**Δ Circulating CD3+CD8+ T Lymphocytes Count**			
Median (Min-max)	-0.08 (-0.39; 1.23)	-0.13 (-0.23; 0.33)	0.62
**Δ Circulating CD4/CD8 Ratio**			
Median (Min-Max)	0.05 (-0.23; 0.48)	0.05 (-0.08; 0.21)	0.62
**Δ Circulating CD19+ B Lymphocytes %**			
Median (Min-max)	-0.04 (-0.57; 0.30)	0.03 (-0.25; 1.83)	0.56
**Δ Circulating CD19+ B Lymphocytes count**			
Median (Min-max)	-0.12 (-0.38; 0.19)	-0.19 (-0.38; 2.50)	0.94
**Δ Circulating CD16+CD56+ NK Lymphocytes %**			
Median (Min-max)	-0.13 (-0.46; 0.64)	-0.05 (-0.48; 0.26)	0.61
**Δ Circulating CD16+CD56+ NK Lymphocytes Count**			
Median (Min-max)	-0.19 (-0.63; 1.03)	-0.09 (-0.63; 0.37)	0.59

Legend. Δ: Delta; Δ formula: (Post-therapy value - Baseline Value)/Baseline Value; SD: standard deviation; TILs: tumor-infiltrating lymphocytes; SUV: standard uptake volume; min: minimum; max: maximum; CD3+CD4+: T helper; CD3+CD8+: T suppressors; CD19+: B lymphocytes; CD16+CD56+: NK lymphocytes; g*BRCA*-wt: germinal *BRCA*1/2-wild type triple negative tumors; g*BRCA*-mut: germinal *BRCA*1/2-mutant tumors. *: exploratory comparisons.

Supplementary table 4. Biomarker variation after olaparib treatment according to clinical and radiometabolic response

Biomarkers variation	Clinical Response		*P*	Radiometabolic Response		*P*
Clinical, pathologic and radiometabolic biomarkers	Responders	Non-responders		Responders	Non-responders	
	N=16 (45.7%)	N=19 (54.3%)		N=15 (55.6%)	N=12 (44.4%)	
**Δ Ki-67**						
Median (Min - max)	0.0 (-0.18; 0.33)	0.0 (-0.20; 1.57)	0.20	0.0 (-0.11; 1.50)	0.06 (-0.20; 1.57)	0.74
**Δ Dimension**						
Mean (SD)	-45.9 (±0.12)	-0.08 (±0.17)	*<0.001*	-0.34 (±0.21)	-0.10 (±0.27)	*0.01*
**Δ SUV**						
Mean (SD)	-0.50 (±0.40)	-0.09 (±0.50)	*0.03*	-0.62 (±0.25)	0.17 (±0.34)	*<0.001*
**Δ TILs**						
Median (Min - max)	0 (-1; 3)	0 (-1; 8)	0.75	0 (0; 3)	0 (-1; 8)	0.92
**Immune Circulating Biomarkers**	N=15 (46.9%)	N=17 (53.1%)		N=12 (50.0%)	N=12 (50.0%)	
**Circulating T-regs at basal**						
Median (Min - max)	82 (30 – 238)	78 (12 – 297)	0.70	121 (29 – 238)	74 (32 - 159)	0.57
**Δ Circulating T-reg**						
Median (Min - max)	-0.17 (-0.48; 0.77)	-0.17 (-0.78; 2.91)	0.87	-0.19 (-0.77; 0.77)	-0.16 (-0.35; 0.65)	0.56
**Δ Circulating CD3+CD4+ T Lymphocytes %**						
Mean (SD)	0.07 (±0.09)	0.06 (±0.07)	0.95	0.08 (±0.10)	0.06 (±0.05)	0.61
**Δ Circulating CD3+CD4+ T Lymphocytes Count**						
Median (Min - max)	0.03 (-0.23; 0.71)	-0.03 (-0.34; 0.27)	0.63	-0.04 (-0.34; 0.71)	-0.03 (-0.32; 0.27)	0.94
**Δ CD3+CD8+ T Lymphocytes %**						
Median (Min - max)	-0.01 (-0.13; 0.48)	0.01 (-0.15; 0.18)	0.99	0.01 (-0.14; 0.48)	-0.05 (-0.15; 0.12)	0.24
**Δ CD3+CD8+ T Lymphocytes Count**						
Median (Min - max)	-0.11 (-0.39; 1.23)	-0.11 (-0.32;0.33)	0.97	-0.06 (-0.39; 1.23)	-0.11 (-0.29; 0.03)	0.44
**Basal CD4+/CD8+ Ratio**						
Median (Min - max)	1.97 (0.94 – 5.94)	3.23 (1.17 – 7.23)	0.16	2.46 (0.94 – 7.23)	2.77 (1.12 – 5.94)	0.54
**Δ CD4+/CD8+ Ratio**						
Mean (SD)	0.06 (±0.17)	0.06 (±0.12)	0.96	0.05 (±0.17)	0.10 (±0.12)	0.39
**Δ T-regs/T-eff ratio**						
Median (Min - max)	-0.09 (-0.39; 0.18)	-0.10 (-0.71; 3.53)	0.98	-0.13 (-0.71; 0.35)	-0.05 (-0.25; 0.70)	0.10
**Δ T-regs/T-eff ratio %**						
Median (Min-Max)	-0.20 (-0.49; 0.40)	-0.20 (-0.78; 2.58)	0.70	-0.18 (-0.78; 0.40)	-0.20 (-0.37; 0.55)	0.56
**Δ CD19+ B Lymphocytes %**						
Median (Min - max)	-0.10 (-0.57; 0.18)	-0.04 (-0.22; 1.83)	0.31	-0.11 (-0.57; 0.17)	-0.03 (-0.15; 0.30)	0.29
**Δ CD19+ B Lymphocytes Count**						
Median (Min - max)	-0.20 (-0.38; 0.23)	-0.11 (-0.37; 2.50)	0.2	-0.18 (-0.38; 0.19)	-0.07 (-0.34; 0.23)	0.35
**Δ CD16+CD56+ NK Lymphocytes %**						
Mean (SD)	-0.10 (±0.28)	-0.11 (±0.17)	0.9	-0.08 (±0.31)	-0.10 (±0.20)	0.86

**Δ CD16+CD56+ NK Lymphocytes Count**						
Median (Min - max)	-0.10 (-0.63; 1.03)	-0.22 (-0.54; 0.61)	0.58	-0.13 (-0.63; 0.61)	-0.19 (-0.45; 0.13)	0.41

Legend. Δ: Delta; Delta Clinical Dimension: (Post-therapy value-Baseline Value)/Baseline Value; Delta Pathological Dimension: (Pathological tumor size at surgery -Baseline Value)/Baseline Value; SD: standard deviation; TILs: tumor-infiltrating lymphocytes; SUV: standard uptake volume; Responders: patients with partial response to olaparib short course; Non-responders: patients with stable disease or progressive disease after olaparib short course; T-regs/T-eff ratio: T.regs count or %/(CD3+CD4+ count or % + CD3+CD8+ count or %).

Supplementary table 5. Basal TILs, T-regs/t-eff ratio and PD-L1 levels according to clinical/radiometabolic responses

**Variables**	**Clinical Responses**				***P***
**Basal PD-L1**	**Responders**		**Non-responders**		
	**N**	**%**	**N**	**%**	
Positive	13	86.7	9	56.3	0.11
Negative	2	13.3	7	43.7	
**Basal TILs**					
Median (Min-max)	90 (10-90)	-	40 (10-90)	-	0.13
**T-regs/T-eff ratio**					
Median (Min-Max)	0.07 (0.03 - 0.14)	-	0.07 (0.01 - 0.14)	-	0.99
**T-regs/T-eff ratio %**					
Median (Min-Max)	1.30 (0.48 - 3.17)	-	1.17 (0.20 - 4.97)	-	0.77
**Variables**	**Radiometabolic Responders**				***P***
**Basal PD-L1**	**Responders**		**Non-responders**		
	**N**	**%**	**N**	**%**	
Positive	12	80.0	7	63.6	0.41
Negative	3	20.0	4	36.4	
**Basal TILs**					
Median (Min-max)	90 (10-90)	-	25 (10-90)	-	0.07
**T-regs/T-eff ratio**					
Median (Min-Max)	0.07 (0.03 - 0.14)	-	0.06 (0.05 - 0.08)	-	0.44
**T-regs/T-eff ratio %**					

Median (Min-Max)	1.70 (0.48 - 3.17)	-	1.13 (0.49 – 2.34)	-	0.57

Legend. TILs: tumor-infiltrating lymphocytes; min: minimum; max: maximum.

Supplementary table 6.

**Variables**	**Pre-olaparib**	**Post-olaparib**	***P****
**CD3+CD4+ T Lymphocytes %**			
Mean (SD)	47.21 (±9.49)	51.00 (±9.81)	*<0.001*
**CD3+CD4+ T Lymphocytes Count (cells/Ul)**			
Mean (SD)	960.64 (±451.14)	956 .36 (±452.96)	0.76
**Circulating T-reg (cells/Ul)**			
Median (min - max)	80.0 (11.5 - 297.0)	66.5 (37.5 - 210.0)	0.12
**CD3+CD8+ T lymphocytes %**			
Median (min - max)	19.0 (9.4 - 70.0)	19.3 (8.1 - 40.9)	0.90
**CD3+CD8+ T lymphocytes Count (cells/Ul)**			
Median (min - max)	361 (144 - 932)	287 (134 - 989)	0.10
**CD4+/CD8+ Ratio**			
Mean (SD)	2.90 (±1.61)	3.26 (±1.89)	*0.02*
**T-regs/T-eff ratio**			
Mean (SD)	0.07 (0.01 - 0.14)	0.06 (0.03 - 0.12)	*0.05*
**T-regs/T-eff ratio%**			
Median (min - max)	1.17 (0.20 - 4.97)	0.89 (0.56 - 2.82)	*0.04*
**CD19+ B Lymphocytes %**			
Mean (SD)	11.11 (±5.34)	10.47 (±6.00)	0.18
**CD19+ B Lymphocytes Count (cells/Ul)**			
Mean (SD)	226.27 (±139.01)	195.03 (±128.39)	*0.003*
**CD16+CD56+ NK Lymphocytes %**			
Mean (SD)	18.73 (±7.84)	16.02 (±6.44)	*0.004*
**CD16+CD56+ NK Lymphocytes Count (cells/Ul)**			
Mean (SD)	366.88 (±182.46)	282.18 (±111.84)	*0.003*

Legend and footnotes. CD3+CD4+: T helper; CD3+CD8+: T suppressors; CD19+: B lymphocytes; CD16+CD56+: NK lymphocytes; T-regs/T-eff ratio: T-regs count or %/(CD3+CD4+ count or % + CD3+CD8+ count or %). *: exploratory comparisons.
